# Book Review

**Published:** 1924-04

**Authors:** 


### Reviews of Books.

**Oral Hygiene.** By J. Sim Wallace, D.Sc., M.D., L.D.S. Pp. vii., 76. London: Baillière, Tindall & Cox. 1923. Price 5s. net.—The subject of Oral Hygiene has been receiving the close attention of many observers for some years past. In this little book the author has placed before his readers his own personal conclusions in a manner admitting of no argument. Many of his views are diametrically opposed to those appertaining at the present time. The physiology of the mouth has never been treated at all fully in the standard text books. Dr. Wallace expounds his views at some length, they will certainly justify their appearance in print if they succeed in focussing attention more closely on this subject, and make the student think for himself. The chapter on " Food and Teeth " contains much interesting matter. With regard to dental caries, the author lays great stress on the fact that the factors governing its production are external to the teeth themselves, and that the calcium content in the tooth structure has nothing whatever to do with the subject. This chapter should call for much discussion and perhaps criticism. Any literature on this fascinating and somewhat obscure subject of oral hygiene is bound to be of interest. This little book is a welcome addition, inasmuch as it calls for careful perusal, and brings forward a number of points that are bound to create a discussion.

**Golden Rules of Dental Mechanics.** By HAROLD OSBORN. Second Edition. Pp. 107. Bristol: John Wright and Sons, Ltd. Price 5s. net.—This little book contains a full and fascinating description of the various processes involved in the Dental Laboratory. It is, of course, well recognised that in order to have a sound practical knowledge of Dental Mechanics the student must apply himself diligently to his practical work in the Dental Workroom. A little volume of this description, with its concise and short account of how to proceed, is a welcome addition to the library of small books in note form, and may well prove of great assistance for reference purposes

**Modern Methods in the Diagnosis and Treatment of Heart Disease.** By FRANCIS HEATHERLEY, M.B., B.S. (Lond.), F.R.C.S. Pp. xi., 199. London: Baillière, Tindall and Cox. 1923. Price 5s.—This little book presents the fundamental facts of modern cardiology, now familiar to most teaching physicians, in a form that is designed to make them easily assimilable by men in general practice. The writer, himself a general practitioner, has been brought into contact with the new ideas about cardiac disease through work done on behalf of the Ministry of Pensions. He has fallen in love with the work, and his book is an enthusiastic exposition of its principles and practice. It closes with a plea that we shall all warmly endorse ; to the effect that men in general practice should be less diffident about publishing their observations on disease. We sincerely trust this seed may find good ground somewhere.

**Asthma.** By FRANK COKE, F.R.C.S. Pp. viii., 260. Bristol: John Wright & Sons Ltd. 1923. Price 15s. net.—In no other disease have our conceptions of causation undergone such a revolution as in asthma during the last decade. Mr. Coke remarks that before anything was known about sensitisation to foreign proteins asthma had been attributed to the proximity of horses or cats, with the result that such an explanation was generally ignored as fallacious or fanciful. He recalls to our notice a remarkable book by Hyde Salter, published in 1868, which anticipates our latest work on sensitisation to animal hairs and describes the dermal reaction. Nowadays protein sensitisation is the first explanation looked for in a case of asthma. The third chapter of this book is devoted to the subject, and describes it simply and clearly, together with the technique for applying dermal tests. Treatment occupies a large part of the volume, and is such as will prove most helpful to the practitioner. The difficult problems of specific protein therapy and desensitisation are recognised fairly and dealt with reasonably. In spite of the comparative novelty of Mr. Coke's studies, he is not carried away by theories, nor is he a mere enthusiast for the *aliquid novi*. He has written a lucid, accurate book based on facts, and illustrated by careful clinical observation and experience. We have to abandon much in older theories and remedies for asthma, and Coke's book on asthma is a valuable guide to newer and more rational methods.

**Chronologia Medica.** By SIR D'ARCY POWER, K.B.E., M.B., and C. J. S. THOMPSON, O.B.E. Pp. iv., 278. London: John Bale, Sons, & Danielsson Ltd. 1923. Price 10s. 6d. net.—This is an interesting hand-list of the chief persons, periods and events in the history of medicine. The information it gives is very brief, and it will serve chiefly as a reminder of dates. But, as the authors say in their Foreword, the addition of a fact or two concerning each epoch, event, or person gives to the chronology a semblance of vitality. Medical editors in particular may be glad of such a reference book on their desks.

**" Magistri Salernitani nondum Cogniti." A contribution to the history of the Medical School of Salerno.** By DOCTOR PIETRO CAPPARONI, with a Foreword by SIR D'ARCY POWER, K.B.E., M.B. (Oxon.), F.R.C.S. Pp. v., 68. London: John Bale, Sons & Danielsson Ltd. 1923. Price 15s. net.—This is the second volume of Research Studies which we owe to the Wellcome Historical Museum, and is of remarkable interest and value. Dr. P. Capparoni, when on military duty at Salerno during the war, met with a manuscript containing a list of 13,000 names of members of certain guilds in the cathedral archives. The first entries were made in the eleventh century, but they include names from still earlier times. From these he collected the names, dates and titles of a number of medical men previously unknown, who taught and practised in the school of Salernum, and also details about others who are mentioned by De Renzi, Henschel and Daremberg, the historians of the school. He has also given us an admirable sketch of the annals of the school and of medical education during its existence. In his view the revival of the medical art about the beginning of the ninth century took place almost entirely in the monastic and episcopal schools. Salernum was one of the last towns to retain its independence and Roman institutions. It contained many Benedictine houses, with their libraries and infirmaries. It was on the direct route to the Byzantine world, and Greek was still spoken in the neighbourhood. Hence the early renaissance of classical medicine here. The earliest doctors were nearly all ecclesiastics, but the interference with their work became so great that in the twelfth century council after council forbade their leaving their duties to study law and medicine. New universities and guilds of lay doctors then appeared to take their place at Salernum and elsewhere and the school continued to flourish till 1250, when it was overshadowed by the University af Naples. It is interesting to note the fine hospitals and the public baths in the town, and that the proper name of the celebrated woman doctor or midwife was Trocta. Trotula was, in fact, the name of her book, as Rogerina was that of the surgical work of Roger. Twenty-seven beautiful photographic plates add to the attractions of this scholarly volume, which is a mine of information about the earliest renaissance of medicine.

**Diseases of the Rectum and Colon.** By C. LOCKHART-MUMMERY, F.R.C.S. Pp. x., 872. London: Baillière, Tindall & Cox. 1923. Price 25s. net.—Dealing with the closely-associated diseases of the rectum and colon, this bock emphasises the importance of the sigmoidoscope in their diagnosis, its value in accurate localisaticn and differentiation of disease in these organs being comparable with that of the cystoscope, which revolutionised the differential diagnosis of urinary diseases. The importance of realising that rectal operations can be rendered relatively aseptic by suitable preparation should encourage efforts in this direction, and eliminate some of the ills resulting from despair of attaining surgical cleanliness in rectal work. While it cannot be regarded as an exhaustive treatise on the subject, diverticulitis is fully dealt with and illustrated by clinical histories and operations and post-mortem findings. The book will be fcund of value to the surgeon with occasional opportunities only of gaining experience in these subjects, and to the general practitioner from the point of view of diagnosis.

**Diseases of the Male Organs of Generation.** By KENNETH M. WALKER, M.A., F.R.C.S., M.B., B.C. Pp. xii., 234. London: Oxford Medical Publications. 1923. Price 12s. 6d. net.—The practitioner and student will find this a useful book, for though not exhaustive, most subjects are adequately dealt with, and some, such as prostatic enlargement, fairly fully. For instance, the author discusses the question whether, as in his opinion, the enlargement is due to fibro-epithelial degenerative changes associated with the end of sexual life and fully comparable with the changes in the female breast which occurs at that time, or, as is usually held, an adenomatous growth. He emphasises the importance of the two-stage operation in prostatectomy and the inadvisability of making the Thompson Walker exposure a routine procedure, with both of which opinions one is in full agreement. The more modern outlook in acute prostatitis and vesiculitis is touched on; the work on internal secretion of the testicles is considered and Steinach's rejuvenation experiments and Voronoff's work on testicular grafts. Venereal disease is deliberately omitted, and pathology also, except where it has a definite bearing on treatment, which is made a particular feature of the book for the benefit of the student and practitioner. It is well worth reading, for it gives evidence of original thought and research.

**An Introduction to Surgical Urology.** By W. K. Irwin, M.D. (Aberd.), F.R.C.S. (Edin.). Pp. vii., 180. London: Baillière, Tindall & Cox. 1923. Price 7s. 6d. net.—This book, as its title implies, will be found of value to those with little experience of genito urinary surgery. To the practitioner and student it introduces the subject from the consulting-room point of view. Commencing with a brief anatomical description of the organs involved, it passes to the methods of inquiry into and examination of the diseases of the urinary tract. The investigations necessary to the elucidation of its prominent signs and symptoms, to each of which a chapter is devoted, then follows, and, finally, enlargement of the prostate is more fully dealt with.

**Incompatibility in Prescriptions and how to Avoid it.** By Thos. Stephenson, D.Sc. Edinburgh: The Prescriber Offices. 1924. 1s. 6d. net.—Dr. Stephenson quite correctly states in his Introduction that the time devoted by medical students to chemistry and pharmacy in their curriculum is short; we consider that it is lamentably short! The result of this is that when later on they attempt to write prescriptions for their patients they frequently find themselves hopelessly involved in their efforts to avoid the various types of incompatibility which are met with in pharmacy. This little monograph will be very useful to such students, and will enable them to avoid mistakes which would not add to their reputation. Dr. Stephenson deals briefly and concisely with the subject, and his wide experience has enabled him to select examples for discussion which are met with in actual practice. The fact that several reprints of the work have been necessary speaks for its utility, and we have every confidence in recommending it to students and junior practitioners of medicine.

